# Real-world use and clinical impact of an electronic patient-reported outcome tool in patients with solid tumors treated with immuno-oncology therapy

**DOI:** 10.1186/s41687-024-00700-4

**Published:** 2024-02-28

**Authors:** Natalie R Dickson, Karen D Beauchamp, Toni S Perry, Ashley Roush, Deborah Goldschmidt, Marie Louise Edwards, L Johnetta Blakely

**Affiliations:** 1 Tennessee Oncology, 2004 Hayes Street – 8th Floor, Nashville, TN 37203 USA; 2 Bristol Myers Squibb, Princeton, NJ USA; 3 Varian Medical Systems, Atlanta, GA USA; 4 Analysis Group Inc, New York NY, USA

**Keywords:** Community oncology practice, Duration of therapy, Electronic medical records, Electronic patient-reported outcome, Health-related quality of life, Immuno-oncology therapy, Overall survival, Real-world, Symptom management, Symptom reporting

## Abstract

**Background:**

Utilization of electronic patient-reported outcome (ePRO) tools to monitor symptoms in patients undergoing cancer treatment has shown clinical benefits. Tennessee Oncology (TO) implemented an ePRO platform in 2019, allowing patients to report their health status online. We conducted a real-world, multicenter, observational, non-interventional cohort study to evaluate utilization of this platform in adults with solid tumors who initiated immuno-oncology (IO) therapy as monotherapy or in combination at TO clinics.

**Methods:**

Patients initiating IO therapy prior to platform implementation were included in a historical control (HC) cohort; those initiating treatment after implementation were included in the ePRO cohort, which was further divided into ePRO users (platform enrollment ≤ 45 days from IO initiation) and non-users. Data were extracted from electronic medical records; patients were followed for up to 6 months (no minimum follow up). Outcomes included patient characteristics, treatment patterns, duration of therapy (DoT), and overall survival (OS).

**Results:**

Data were collected for 538 patients in the HC and 1014 in the ePRO cohort; 319 in the ePRO cohort were ePRO users (uptake rate 31%). Baseline age was higher, more patients had stage IV disease at diagnosis, and more received monotherapy (82 vs 52%, respectively) in the HC vs the ePRO cohort. Median follow-up was 181.0 days (range 0.0–182.6) in the HC and 175.0 (0.0–184.0) in the ePRO cohort. Median DoT of index IO regimen was 5.1 months (95% confidence interval [CI], 4.4–NE) in the HC cohort vs not estimable (NE) in the ePRO cohort. Multivariable regression adjusting for baseline differences confirmed lower risk of treatment discontinuation in the ePRO vs HC cohort: hazard ratio (HR) 0.83 (95% CI, 0.71–0.97); *p* < 0.05. The estimated 6-month OS rate was 65.5% in the HC vs 72.4% in the ePRO cohort (*p* < 0 .01). Within the ePRO cohort, DoT of index IO regimen and OS did not differ between users and non-users. In ePRO users, patient platform use was durable over 6 months.

**Conclusion:**

Improvements in DoT and OS were seen after ePRO platform implementation. Conclusions are limited by challenges in separating the impact of platform implementation from other changes affecting outcomes.

**Supplementary Information:**

The online version contains supplementary material available at 10.1186/s41687-024-00700-4.

## Background

Through improvements in treatments and technologies, many cancers have become long-term chronic conditions rather than acute diseases with poor survival. Cancer treatments have also become more complex, thus symptom management has become an increasingly challenging and important component of care. Patients undergoing cancer treatment frequently experience disease- and treatment-related symptoms, which are often underreported and underestimated by physicians [1–6]. The reporting process usually relies on a physician’s interpretation of a patient’s recollection of symptoms or adverse events, which may result in inaccuracies [7]. Furthermore, patients may not be well-informed regarding which symptoms require urgent medical attention [8]. In patients receiving active treatment, patient-reported symptom information is important to inform treatment decisions; an increase in symptom burden may also indicate disease progression [9]. Assessment of toxicity helps guide decisions on prevention and management (eg, dose reductions/delays), and may improve outcomes by enabling patients to tolerate therapy for longer [10].

Immuno-oncology (IO) therapies have improved clinical outcomes in multiple cancers; however, these agents have toxicity profiles related to their mode of action that differ from those of standard treatments such as chemotherapy [11, 12]. These unique immune-related adverse events (irAEs) may have prolonged duration, delayed onset, and onset after treatment discontinuation [11, 12]. Timely identification of irAEs is important to allow rapid management of toxicities and improve symptom resolution, which could result in patients remaining on treatment for longer with fewer dose interruptions.

Utilization of electronic patient-reported outcome (ePRO) tools to monitor symptoms in clinical trials enrolling patients with cancer have shown clinical benefit regarding symptom-related distress, health-related quality of life, healthcare resource utilization (HCRU), and overall survival [13–15]. Real-world data also support a positive impact of ePRO use on HCRU [16–18]. The reasons for improved outcomes with ePROs are multifaceted and likely to involve a combination of improved communication between healthcare provider and patient, a move towards ‘whole’ person care resulting in better treatment of each individual’s needs, early detection of symptoms, enhanced monitoring of treatment efficacy, and increased patient engagement, which can contribute to increased adherence to treatment plans [18–22]. Overall, the result is enhanced, patient-centered care which seemingly improves patient outcomes.

While increasingly used in clinical trials, ePRO tools have not yet been widely adopted in community oncology practices [23–26]. In 2018, Tennessee Oncology (TO), one of the largest community-based cancer care practices in the US, initiated development of a customized electronic patient care coordination platform to allow patient-reported symptoms to be captured easily and enable triage and care coordination teams to process information in real time. Previously, ‘treatment callbacks’ following each round of therapy were an integral component of care to help manage patient symptoms and increase clinical intervention; these were time-consuming, and clinic staff may not have had capacity to make multiple call attempts [27]. Additionally, it was hoped that an ePRO tool would allow patient status information to be readily accessible and directly linked to patient records, whereas information in the electronic medical record (EMR) cannot necessarily be accessed by the care team quickly and easily, particularly when data are not captured in discrete fields. The ePRO platform was implemented by TO in 2019 to allow patients to easily report their health status and allow the healthcare team to proactively contact patients at scheduled times to provide prompt symptom management. The platform has since become the single electronic interface at TO for all patient communication activities. We conducted this real-world study to evaluate use of the ePRO platform and its impact on treatment patterns, most importantly duration of therapy (DoT), as well as overall survival (OS), in patients with solid tumors receiving IO therapy in community practice.

## Methods

This was a real-world, multicenter, observational, non-interventional cohort study. The study population comprised adults (≥18 years of age at index date) with a documented diagnosis of solid tumor malignancy (non-small cell lung cancer [NSCLC], melanoma, renal cell carcinoma, bladder cancer, or head and neck cancer) who initiated IO therapy as monotherapy or in combination with any other therapy at TO clinics. IO therapy included atezolizumab, avelumab, durvalumab, ipilimumab, nivolumab, or pembrolizumab; other IO therapies that received approval after the study launch date for the specified tumor types were also considered. Exclusion criteria were enrollment in any clinical trial or pregnancy at index.

**Fig. 1:**
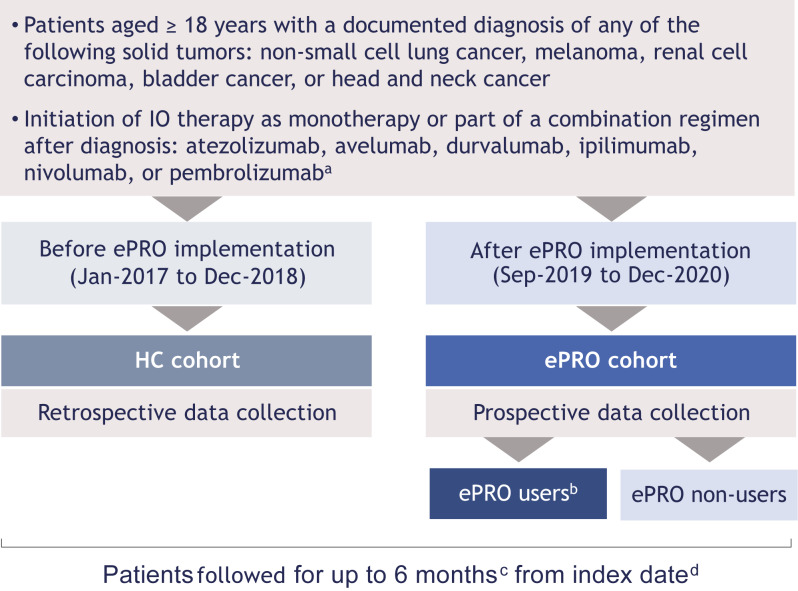
Study design. ^a^In the ePRO cohort, other IO therapies indicated for the solid tumors of interest could be considered after launch of the study. ^b^Voluntary enrollment in the ePRO platform within 45 days of index date. ^c^Until death, loss to follow-up, pregnancy, enrollment in a clinical trial, or 6 months post-IO therapy initiation. ^d^Date on which the first cycle of IO therapy was initiated after diagnosis. *ePRO* electronic patient-reported outcome; *HC* historical control; *IO* immuno-oncology

The study design is shown in Fig. 1. Study objectives were to describe and compare patient characteristics, treatment patterns, DoT, and OS in patients receiving IO before vs after ePRO platform implementation, and to evaluate use of the platform during the post-implementation period. Patients initiating IO therapy immediately before implementation of the ePRO tool (between January 1, 2017 and December 31, 2018) were included in the historical control (HC) cohort. Those initiating IO therapy after implementation (between September 1, 2019 and December 31, 2020) were included in the ePRO cohort. Patients in the ePRO cohort were invited to use the ePRO platform and could either accept or decline. Thus, the ePRO cohort was further divided into ePRO users (those accepting to use the platform within 45 days of the index date) and non-users. The index date for both the HC and ePRO cohorts was the date that the first cycle of IO therapy was initiated. All patients were followed for up to 6 months from index date until death, loss to follow-up, pregnancy, enrollment in a clinical trial, or end of the 6-month follow-up period; there was no minimum follow-up period.

De-identified patient-level data were extracted from the TO EMR database for the HC and ePRO cohorts; additional data for the ePRO users subgroup were exported from the ePRO platform. Data for the HC cohort were collected retrospectively; data for the ePRO cohort were collected prospectively at regular intervals. Abstraction of data from EMRs was conducted by designated full-time employees at TO; data were then provided to Analysis Group for analysis.

### EPRO platform

The ePRO platform implemented by TO was the Noona® patient outcomes management solution (Varian Medical Systems). Noona is a cloud-based, scientifically validated, mobile platform designed to capture PROs in routine clinical practice, allowing patients to report their health status online. Healthcare providers can interact with the platform online or at the clinic and respond to patient communications. Training for physicians and nurse practitioners was conducted at the 33 TO clinics. Care coordinators and other support staff who would use the platform received intensive training and continued to receive ongoing support. Patients also received training and support. Along with notifications sent to alert patients about the change, front-office staff supported patients in setting up online accounts. Patients received hands-on support to allay concerns and address any technological challenges. For ePRO users, questionnaires based on the PRO-Common Terminology Criteria for Adverse Events (PRO-CTCAE) were sent within a week of each IO infusion and could be completed using an internet browser or tablet/smartphone app. PRO-CTCAE was utilized as TO were familiar with this instrument, allowing adoption of the new ePRO platform alongside a known PRO instrument, meaning minimal disruption to typical routines.

### Data collection

Demographic, clinical, and disease characteristics were described for each cohort; computer literacy was also described for ePRO users and non-users based on patient self-report at index visit. Treatment patterns (number of regimens, monotherapy vs combination therapy, therapy type) were described for each cohort. DoT was defined as time from index date until end of treatment and was assessed for the index IO regimen and the subsequent treatment regimen. Index IO regimen was defined as the treatment that included the patient’s first IO therapy for the index cancer (which may not be the patient’s first overall line of therapy for index cancer). The IO component could start after the beginning of the overall regimen, and/or end before the end of the overall regimen. The subsequent regimen was defined as the next treatment after index IO regimen (may or may not include an IO agent). For both index and subsequent regimens, treatment was considered to have ended when all agents in that regimen were stopped, as defined in patient charts, although the same chemotherapy may have been maintained across regimens. OS was measured from index date until date of death or censoring. The proportion of patients in the ePRO cohort who opted to use the ePRO platform was described, along with the level of patient/healthcare provider use of the platform in the ePRO users subgroup. Assessments were receipt of questionnaires, response to questionnaires, reported symptoms, alerts based on symptoms, and durability of use over time. Outcomes were assessed for two consecutive 3-month periods, Months 1–3 and Months 4–6, with month 1 starting on the index date.

### Statistical analysis

Statistical analysis was performed using SAS Enterprise Guide 7.1 software (SAS Institute, North Carolina) and R (The R Foundation). All outcomes were analyzed descriptively and compared between the HC and ePRO cohorts; comparisons between ePRO users and non-users were also made for demographic and clinical characteristics, computer literacy, DoT, OS, and ePRO platform usage. Continuous variables were described using mean, standard deviation (SD), and median, and compared with the Wilcoxon rank sum test; categorical variables were described with frequencies and proportions and compared using the chi-squared test.

Kaplan–Meier analyses were conducted for DoT and OS; patients were censored at end of follow-up (6 months after index) or if they died, became pregnant, or enrolled in a clinical trial at the last contact date with TO. For DoT analysis, patients who had a dose interruption or treatment holiday were considered still on treatment if the treatment plan had not changed. Landmark analysis was conducted for DoT and OS to determine the proportion of patients still on therapy/alive at 1-month intervals after treatment initiation. A univariable Cox proportional hazards model was used to compare DoT and OS in the HC and ePRO cohorts. Additionally, multivariable fitted Cox proportional hazards regression models were used to adjust for differences in baseline characteristics between the cohorts in the DoT and OS analyses and included the covariates of cohort, age at index, sex, race, index cancer, and index cancer stage at diagnosis. Multivariable analysis for DoT comparing the ePRO users and non-users subgroups also included the covariate of insurance type.

To explore the potential impact of differences in treatment patterns, the same analyses were conducted comparing DoT and OS in the subgroup of all patients who received monotherapy for first IO regimen with all those receiving combination therapy for first IO regimen (from the combined HC and ePRO cohorts). The multivariable analysis included the covariates used for the HC and ePRO comparison plus insurance type and index year.

## Results

Data were collected for 538 patients in the HC cohort and 1014 patients in the ePRO cohort. In the ePRO cohort, 319 patients opted to use the ePRO platform and so became ePRO users while 695 were non-users (ePRO uptake rate of 31%).

Demographic and clinical characteristics are shown in Table 1. Patients in the HC cohort were older at baseline than those in the ePRO cohort (70.2 vs 68.3 years; *p* < 0.01). More patients in the ePRO cohort had a college/graduate degree level of education (16.3 vs 4.5%; *p* < 0.001), though this information was missing for 55.6% of patients in the ePRO cohort and 88.8% in the HC cohort. A greater proportion of patients in the HC vs the ePRO cohort had stage IV disease (54.3 vs 47.0%; *p* < 0.01), had metastatic recurrence in those diagnosed with stage I to III cancer (61.8 vs 39.1%; *p* < 0.001), had a greater number of prior lines of therapy (mean 0.6 [SD 0.7] vs 0.3 [0.5], respectively; *p* < 0.001), and died during the study follow-up period (34.4 vs 26.9%, respectively; *p* < 0.01). Differences were also observed between the HC and ePRO cohorts in index cancer type, index IO therapy, and reason for end of follow-up (all *p* ≤ 0.01).

**Table 1:** Demographic, disease, and treatment characteristics in the HC and ePRO (users and non-users) cohorts

Parameter	HC cohort (n = 538)	ePRO cohort (n = 1014)	*P* value^a^	ePRO cohort		*P* value^a^
				ePRO users (n = 319)	ePRO non-users (n = 695)	
Age at index date, years, mean (SD)	70.2 (11.0)	68.3 (10.7)	< 0.01	67.8 (10.6)	68.6 (10.7)	0.220
Female, n (%)	205 (38.1)	405 (39.9)	0.515	145 (45.5)	260 (37.4)	< 0.05
Race, n (%)						
White	484 (90.0)	897 (88.5)	0.416	299 (93.7)	598 (86.0)	< 0.001
Black or African American	41 (7.6)	93 (9.2)	0.347	12 (3.8)	81 (11.7)	< 0.001
Asian	2 (0.4)	4 (0.4)	1.000	2 (0.6)	2 (0.3)	0.594
American Indian or Alaska Native	1 (0.2)	5 (0.5)	0.671	2 (0.6)	3 (0.4)	0.652
Native Hawaiian or Other Pacific Islander	0 (0.0)	2 (0.2)	0.547	1 (0.3)	1 (0.1)	0.530
Mixed race	1 (0.2)	0 (0.0)	0.347	0 (0.0)	0 (0.0)	–
Unknown^b^	9 (1.7)	15 (1.5)	0.830	3 (0.9)	12 (1.7)	0.413
Ethnicity, n (%)			0.131			0.464
Hispanic or Latino	4 (0.7)	8 (0.8)		1 (0.3)	7 (1.0)	
Not Hispanic or Latino	492 (91.4)	895 (88.3)		280 (87.8)	615 (88.5)	
Unknown	42 (7.8)	111 (10.9)		38 (11.9)	73 (10.5)	
Type of insurance,^c^ n (%)			0.120			< 0.001
Non-risk-share contracts	316 (58.7)	638 (62.9)		161 (50.5)	477 (68.6)	
Risk-share contracts^d^	222 (41.3)	376 (37.1)		158 (49.5)	218 (31.4)	
Highest level of education,^c^ n (%)			< 0.001			< 0.001
High school or less	36 (6.7)	285 (28.1)		88 (27.6)	197 (28.3)	
College	18 (3.3)	124 (12.2)		54 (16.9)	70 (10.1)	
Graduate degree	6 (1.1)	41 (4.0)		24 (7.5)	17 (2.4)	
Unknown	478 (88.8)	564 (55.6)		153 (48.0)	411 (59.1)	
Marital status,^c^ n (%)			0.173			< 0.001
Married	311 (57.8)	596 (58.8)		222 (69.6)	374 (53.8)	
Divorced	81 (15.1)	119 (11.7)		26 (8.2)	93 (13.4)	
Widowed	77 (14.3)	153 (15.1)		38 (11.9)	115 (16.5)	
Single	61 (11.3)	133 (13.1)		31 (9.7)	102 (14.7)	
Separated	1 (0.2)	7 (0.7)		1 (0.3)	6 (0.9)	
Unknown	7 (1.3)	6 (0.6)		1 (0.3)	5 (0.7)	
Living arrangements,^c^ n (%)			0.183			< 0.001
With spouse	301 (55.9)	585 (57.7)		218 (68.3)	367 (52.8)	
Alone	99 (18.4)	219 (21.6)		50 (15.7)	169 (24.3)	
With child	46 (8.6)	75 (7.4)		17 (5.3)	58 (8.3)	
With relatives	44 (8.2)	58 (5.7)		11 (3.4)	47 (6.8)	
Care facility	5 (0.9)	11 (1.1)		0 (0.0)	11 (1.6)	
Other	26 (4.8)	48 (4.7)		15 (4.7)	33 (4.7)	
Unknown	17 (3.2)	18 (1.8)		8 (2.5)	10 (1.4)	
Index cancer,^e^ n (%)			< 0.01			< 0.01
NSCLC	351 (65.2)	697 (68.7)		211 (66.1)	486 (69.9)	
Melanoma	101 (18.8)	124 (12.2)		54 (16.9)	70 (10.1)	
Renal cell carcinoma	34 (6.3)	74 (7.3)		17 (5.3)	57 (8.2)	
Head and neck cancer	34 (6.3)	62 (6.1)		15 (4.7)	47 (6.8)	
Bladder cancer	18 (3.3)	57 (5.6)		22 (6.9)	35 (5.0)	
Stage of index cancer at diagnosis, n (%)			< 0.01			0.429
Stage I	33 (6.1)	69 (6.8)		21 (6.6)	48 (6.9)	
Stage II	57 (10.6)	82 (8.1)		21 (6.6)	61 (8.8)	
Stage III	122 (22.7)	327 (32.2)		100 (31.3)	227 (32.7)	
Stage IV	292 (54.3)	477 (47.0)		162 (50.8)	315 (45.3)	
Unknown	34 (6.3)	59 (5.8)		15 (4.7)	44 (6.3)	
Progression since diagnosis,^f^ among patients diagnosed with stage I to III, n (%)			< 0.001			0.277
Yes, metastatic recurrence	131 (61.8)	187 (39.1)		57 (40.1)	130 (38.7)	
Yes, local/regional recurrence	39 (18.4)	55 (11.5)		11 (7.7)	44 (13.1)	
No	42 (19.8)	232 (48.5)		72 (50.7)	160 (47.6)	
Unknown	0 (0.0)	4 (0.8)		2 (1.4)	2 (0.6)	
Number of lines of prior therapy for index cancer,^g,h^ mean (SD)	0.6 (0.7)	0.3 (0.5)	< 0.001	0.3 (0.5)	0.3 (0.5)	0.363
Index IO therapy,^e^ n (%)			< 0.001			0.603
Atezolizumab	31 (5.8)	38 (3.7)		13 (4.1)	25 (3.6)	
Avelumab	0 (0.0)	2 (0.2)		1 (0.3)	1 (0.1)	
Durvalumab	1 (0.2)	221 (21.8)		65 (20.4)	156 (22.4)	
Ipilimumab	16 (3.0)	3 (0.3)		0 (0.0)	3 (0.4)	
Nivolumab	192 (35.7)	115 (11.3)		43 (13.5)	72 (10.4)	
Nivolumab + ipilimumab	20 (3.7)	86 (8.5)		29 (9.1)	57 (8.2)	
Pembrolizumab	278 (51.7)	549 (54.1)		168 (52.7)	381 (54.8)	
Time to the end of follow-up (days),	181.0	175.0	0.678	175.0	175.0	0.742
median (range)	(0, 182.6)	(0, 184.0)		(0, 184.0)	(0, 184.0)	
Mortality during the study follow-up, n (%)			< 0.01			0.107
Alive	343 (63.8)	731 (72.1)		242 (75.9)	489 (70.4)	
Deceased	185 (34.4)	273 (26.9)		76 (23.8)	197 (28.3)	
Unknown	10 (1.9)	10 (1.0)		1 (0.3)	9 (1.3)	
Reason for the end of follow-up (earliest event),^i^ n (%)			< 0.001			< 0.01
Last contact with TO	198 (36.8)	637 (62.8)		219 (68.7)	418 (60.1)	
6-month follow-up	316 (58.7)	340 (33.5)		85 (26.6)	255 (36.7)	
Clinical trial enrollment	18 (3.3)	25 (2.5)		11 (3.4)	14 (2.0)	
Death	6 (1.1)	12 (1.2)		4 (1.3)	8 (1.2)	

^a^Statistical comparison performed for continuous variables using Wilcoxon rank-sum test and for categorical variables using chi-squared test or Fisher’s exact tests if expected counts <10

^b^Patient declined, or otherwise not documented/unknown

^c^Collected from the EMR on the date of abstraction and may not reflect the status at the index date

^d^Risk-share contracts include Medicare, Aetna, and Cigna (for patients who initiated IO therapy after Apr 01, 2020)

^e^The index cancer was defined as the cancer associated with the index IO therapy and the index IO therapy was defined as the IO therapy/therapies initiated on the index date

^f^Progression reported from diagnosis until end of follow-up, as documented by the treating physician was assessed among the 690 patients diagnosed with stage I, II, or III for their index cancer

^g^Summary statistics for the number of lines of prior therapy were assessed among patients with known information

^h^Prior lines of therapy before IO initiation may have occurred at TO or another facility and patients may have had more than one type of therapy

^i^Reason for end of follow-up was defined as the earliest of the following events, if applicable: 6 months after IO initiation, death, pregnancy, clinical trial enrollment, or last contact with TO

*EMR* electronic medical record; *ePRO* electronic patient-reported outcome; *HC* historical control; *IO* immuno-oncology; *NSCLC* non-small cell lung cancer; *SD* standard deviation; *TO* Tennessee Oncology

In the ePRO cohort, ePRO users were more likely than non-users to be female, White, married, living with a spouse, and have a college or graduate degree (all *p* < 0.05), and a greater proportion of patients covered by risk-share contracts were ePRO users (*p* < 0.001). Differences were also observed between ePRO users and non-users in index cancer type and reason for end of follow-up (*p* < 0.01).

Demographic and clinical characteristics for the subgroups of patients receiving monotherapy (n = 969) or combination therapy (n = 583) as first IO regimen are shown in Supplementary Table S1.

In the ePRO cohort, patient and caregiver access to a computer, tablet, or smartphone and frequency of patient email use were unknown in most cases (Table 2) but confirmed access and email use were more common among ePRO users than non-users (all *p* < 0.001).

**Table 2:** Computer literacy in the ePRO cohort (users vs non-users)

Parameter	ePRO users (n = 319)	ePRO non-users (n = 695)	*P* value^a^
Patient access to a computer, tablet, or smartphone, n (%)			< 0.001
Yes	141 (44.2)	184 (26.5)	
No	15 (4.7)	82 (11.8)	
Unknown	163 (51.1)	429 (61.7)	
Caregivers access to a computer, tablet, or smartphone, n (%)			< 0.001
Yes	63 (19.7)	87 (12.5)	
No	5 (1.6)	42 (6.0)	
Unknown	251 (78.7)	566 (81.4)	
Frequency of patient’s email use, n (%)			< 0.001
Daily	80 (25.1)	91 (13.1)	
Weekly	26 (8.2)	21 (3.0)	
Other	27 (8.5)	60 (8.6)	
Never	10 (3.1)	71 (10.2)	
Unknown	176 (55.2)	452 (65.0)	

^a^Statistical comparisons were performed using chi-squared tests (or Fisher’s exact tests for categorical variables with expected counts <10)

*ePRO* electronic patient-reported outcome

The median time to end of follow-up was 181.0 days (range 0.0–182.6) in the HC cohort and 175.0 (range 0.0–184.0) in the ePRO cohort.

### Treatment patterns

The mean (SD) number of regimens from index was 1.1 (0.4) and 1.1 (0.3) in the HC and the ePRO cohorts, respectively. For their index IO regimen, most patients in the HC cohort received monotherapy (82.2% vs combination therapy 17.8%), whereas in the ePRO cohort approximately half received monotherapy (52.0%) and half combination therapy (48.0%). Use of chemotherapy as part of the index regimen was also more frequent in the ePRO than the HC cohort (37.1 vs 13.6%, respectively), as was use of targeted therapy drugs (3.1 vs 0.2%, respectively). For the subsequent regimen, the proportion of patients receiving combination therapy was similar in the HC and ePRO cohorts (57.6 vs 61.9%, respectively). The subsequent regimen included IO therapy in 15.2% of patients in the HC cohort (10 of 66 patients with data) and 25.0% in the ePRO cohort (21 of 84 with data).

### Duration of therapy

**Fig. 2:**
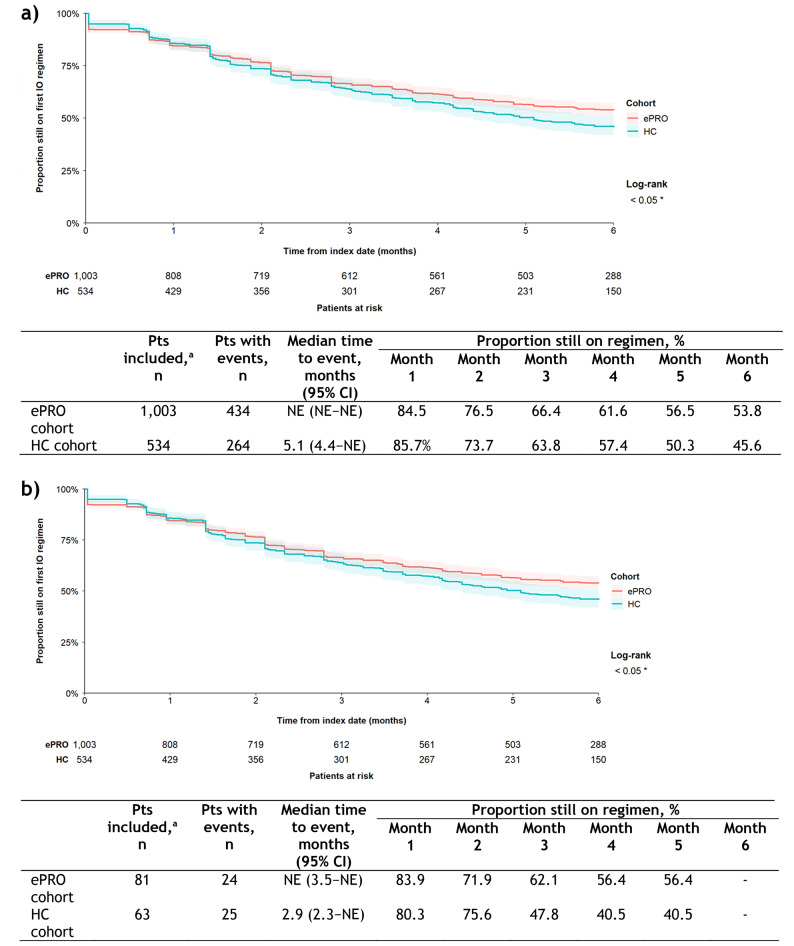
Duration of therapy in HC vs ePRO cohort. **a** index IO regimen and **b** subsequent regimen. ^a^Patients whose end of follow-up date (eg, date of last contact with TO was on their index date were excluded from the analytical subset (no observed follow-up time), as were patients with unknown line of therapy start or end dates. Kaplan-Meier curves were truncated at 6 months from the index date, the maximum observation period. Shaded areas around the curves represent 95% CIs. CI, confidence interval; *ePRO* electronic patient-reported outcome; *HC* historical control; *IO* immuno-oncology; *NE* not estimable; *Pts* patients; *TO* Tennessee oncology

DoT of the index IO regimen was longer in the ePRO cohort than in the HC cohort (Fig. 2a), with median time to end of index IO regimen not estimable (NE) compared with 5.1 months (95% confidence interval [CI], 4.4–NE), respectively. More patients in the ePRO than HC cohort remained on their index IO regimen at every timepoint (6 months: 53.8 vs 45.6%, respectively). For the subsequent regimen, DoT was again longer in the ePRO cohort (n = 81) than in the HC cohort (n = 63), but the difference was not significant (Fig. 2b). Median time to end of therapy was NE in the ePRO cohort (95% CI, 3.5–NE) compared with 2.9 months (2.3–NE) in the HC cohort. The proportion of patients still on their subsequent regimen at 5 months was 56.4% in the ePRO cohort vs 40.5% in the HC cohort. Univariable analysis showed a lower risk of index IO regimen being discontinued in the ePRO vs HC cohort (HR 0.83 [95% CI, 0.71–0.97]; *p* < 0.05); there was no difference regarding risk of subsequent regimen discontinuation (HR 0.81 [95% CI, 0.46–1.41]; *p* = 0.452). Multivariable regression analysis confirmed the univariable findings for index IO regimen, showing a lower risk of treatment discontinuation in the ePRO vs HC cohort (Table 3). There was a higher risk of discontinuation in the index IO regimen in patients with NSCLC (*p* < 0.05) and ‘other’ cancers (*p* < 0.001) vs melanoma, and a lower risk in patients with stage III vs stage I cancer at diagnosis (*p* < 0.05). There were no differences for the subsequent regimen (Table 3).

**Table 3:** Multivariable fitted cox proportional hazards model for dot

Cohort comparison	Regimen	Variate	HR (95% CI)	*P* value
ePRO vs HC^a^	First IO	Cohort (ePRO vs HC)	0.83 (0.71–0.97)	< 0.05
		Age at index date (years)	1.00 (0.99–1.01)	0.781
		Male vs female	1.06 (0.91–1.24)	0.428
		White vs non-White	1.15 (0.90–1.48)	0.259
		Index cancer vs melanoma^b^		
		NSCLC	1.37 (1.07–1.76)	< 0.05
		Other	1.85 (1.41–2.44)	< 0.001
		Stage of index cancer at diagnosis vs stage I		
		Stage II	0.92 (0.64–1.33)	0.668
		Stage III	0.71 (0.52–0.97)	< 0.05
		Stage IV	0.92 (0.69–1.24)	0.587
		Unknown	1.02 (0.68–1.51)	0.942
ePRO vs HC^a^	Second	Cohort (ePRO vs HC)	0.89 (0.50–1.58)	0.685
		Age at index date (years)	0.99 (0.96–1.02)	0.646
		Male vs female	1.29 (0.69–2.41)	0.433
		White vs non-White	0.69 (0.29–1.64)	0.400
		Index cancer vs melanoma^b^		
		NSCLC	2.75 (0.93–8.11)	0.067
		Other	2.11 (0.66–6.74)	0.206
		Stage of index cancer at diagnosis vs stage I		
		Stage II	0.72 (0.20–2.62)	0.617
		Stage III	0.47 (0.14–1.58)	0.223
		Stage IV	0.46 (0.15–1.42)	0.180
		Unknown	0.43 (0.09–2.08)	0.293
ePRO users vs non-users^a^	First IO	Cohort (ePRO users vs non-users)	1.00 (0.81–1.23)	0.973
		Age at index date (years)	1.00 (0.99–1.01)	0.715
		Male vs female	0.95 (0.78–1.16)	0.628
		White vs non-White	1.19 (0.87–1.63)	0.277
		Insurance type (RSC vs non-RSC)	1.02 (0.83–1.25)	0.878
		Index cancer vs melanoma^b^		
		NSCLC	1.27 (0.91–1.77)	0.159
		Other	1.67 (1.16–2.41)	<0.01
		Stage of index cancer at diagnosis vs stage I		
		Stage II	0.83 (0.52–1.33)	0.442
		Stage III	0.61 (0.42–0.90)	<0.05
		Stage IV	0.92 (0.65–1.32)	0.666
		Unknown	0.99 (0.60–1.63)	0.982
ePRO users vs non-users^a^	Second	Cohort (ePRO users vs non-users)	0.30 (0.09–0.94)	< 0.05
		Age at index date (years)	0.99 (0.94–1.05)	0.811
		Male vs female	1.04 (0.38–2.84)	0.941
		White vs non-White	1.00 (0.20–5.00)	1.000
		Insurance type (RSC vs non-RSC)	1.49 (0.49–4.49)	0.481
		Index cancer vs melanoma^b^		
		NSCLC	5.87 (0.72–47.64)	0.097
		Other	3.91 (0.47–32.59)	0.207
		Stage of index cancer at diagnosis vs stage I		
		Stage II	1.21 (0.15–9.41)	0.858
		Stage III	0.34 (0.07–1.64)	0.177
		Stage IV	0.39 (0.09–1.68)	0.205
		Unknown	0.24 (0.02–2.95)	0.266
Monotherapy vs combination therapy	First IO	Regimen (monotherapy vs combination therapy)	0.99 (0.84–1.18)	0.932
		Age at index date (years)	1.00 (0.99–1.01)	0.682
		Male vs female	1.06 (0.91–1.24)	0.448
		White vs non-White	1.15 (0.90–1.47)	0.267
		Insurance type (RSC vs non-RSC)	1.04 (0.89–1.22)	0.630
		Index cancer vs melanoma^b^		
		NSCLC	1.37 (1.07–1.76)	< 0.05
		Other	1.84 (1.40–2.42)	< 0.001
		Index year (n [%]) vs 2017		
		2018	1.19 (0.87–1.63)	0.281
		2019	0.85 (0.66–1.08)	0.182
		2020	0.86 (0.72–1.03)	0.103
		Stage of index cancer at diagnosis vs stage I		
		Stage II	0.91 (0.63–1.31)	0.623
		Stage III	0.71 (0.52–0.97)	< 0.05
		Stage IV	0.92 (0.69–1.23)	0.576
		Unknown	1.02 (0.69–1.53)	0.905

^a^The proportional hazards assumption does not hold for the univariable and multivariable regressions, and results should be interpreted with caution

^b^Melanoma was selected as the reference by the analysis software (first tumor when listed alphabetically)

*CI* confidence interval; *DoT* duration of therapy; *ePRO* electronic patient-reported outcome; *HC* historical control; *HR* hazard ratio; *IO* immuno-oncology; *NSCLC* non-small cell lung cancer; *RSC* risk-share contract

**Fig. 3:**
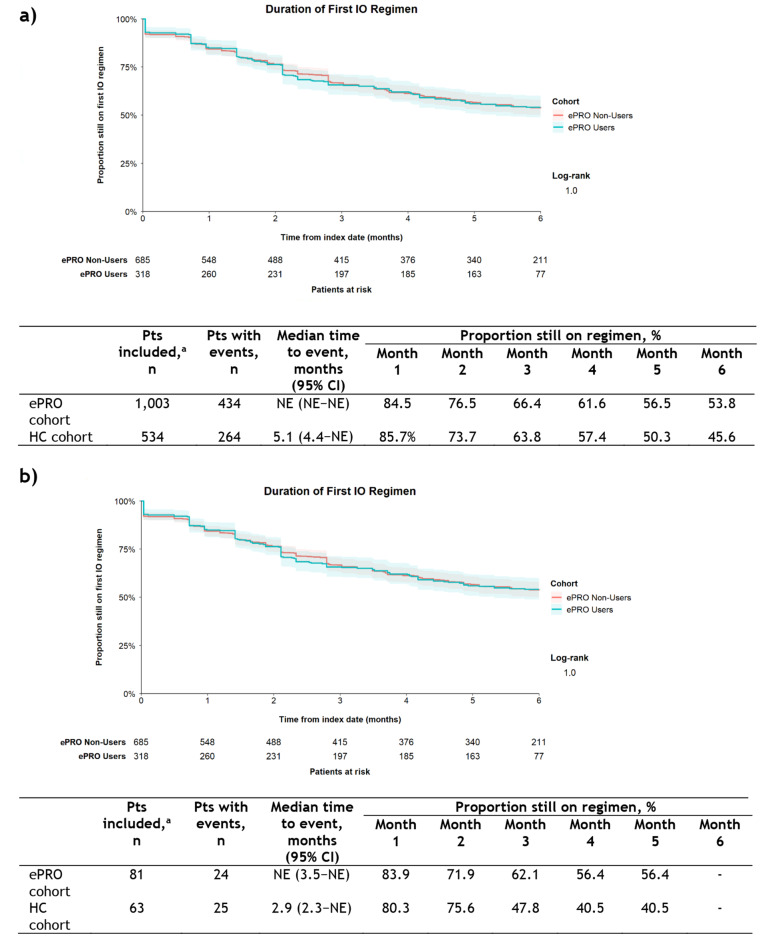
Duration of therapy in ePRO cohort users vs non-users. **a** index IO regimen and **b** subsequent regimen. ^a^Patients whose end of follow-up date (eg, date of last contact with TO) was on their index date were excluded from the analytical subset (no observed follow-up time), as were patients with unknown line of therapy start or end dates. Kaplan-Meier curves were truncated at 6 months from the index date, the maximum observation period. Shaded areas around the curves represent 95% CIs. *CI* confidence interval; *ePRO* electronic patient-reported outcome; *IO* immuno-oncology; *NE* not estimable; *Pts* patients; *TO* Tennessee oncology

Comparison of the ePRO users and non-users subgroups showed no difference in DoT of the index IO regimen (Fig. 3a), with a median time to the end of index IO regimen of NE in both subgroups and the proportion of patients still on index IO regimen at 6 months of 54.1 and 53.7%, respectively. For the subsequent regimen, DoT was longer among ePRO users (n = 29) than non-users (n = 52) but the difference was not significant (Fig. 3b); median time to end of therapy was not reached in either group and the proportion of patients still on the subsequent regimen at 4 months was 63.9 vs 54.7%, respectively. Univariable analysis showed no difference in the risk of either the index IO regimen (HR 1.00 [95% CI, 0.82–1.22]; *P* = 0.997) or subsequent regimen (HR 0.42 [95% CI, 0.15–1.12]; *P* = 0.081) being ended for ePRO users vs non-users. For the index IO regimen, multivariable regression analysis confirmed the findings of the univariable analysis, showing no difference in the risk of treatment being ended between ePRO users and non-users (Table 3). There were effects according to index cancer and index cancer stage at diagnosis for the index IO regimen. For the subsequent regimen, multivariable modeling showed a lower risk of the subsequent regimen being discontinued among ePRO users vs non-users (Table 3).

**Fig. 4:**
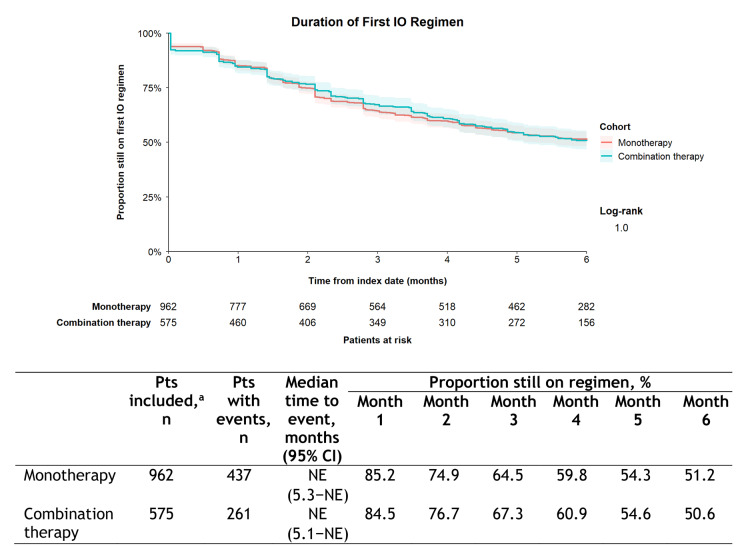
Duration of therapy for first IO regimen in the monotherapy and combination therapy subgroups. ^a^Patients whose end of follow-up date (eg, date of last contact with TO) was on their index date were excluded from the analytical subset (no observed follow-up time), as were patients with unknown line of therapy start or end dates. Kaplan-Meier curves were truncated at 6 months from the index date, the maximum observation period. Shaded areas around the curves represent 95% CIs. *CI* confidence interval; *IO* immuno-oncology; *NE* not estimable; *Pts* patients; *TO* Tennessee oncology

The subgroup analysis comparing DoT in all patients receiving monotherapy with all those receiving combination therapy as first IO regimen showed no difference (Fig. 4), with median time to end of the index IO regimen being NE in both subgroups and proportion of patients still on the index IO regimen at 6 months being 51.2 and 50.6%, respectively (*p* = 1.0). Univariable (HR 1.00 [95% CI, 0.86–1.17]; *p* = 0.990) and multivariable analyses also showed no difference (Table 3). In the latter there were effects according to index cancer type and index cancer stage at diagnosis.

### Overall survival

**Fig. 5:**
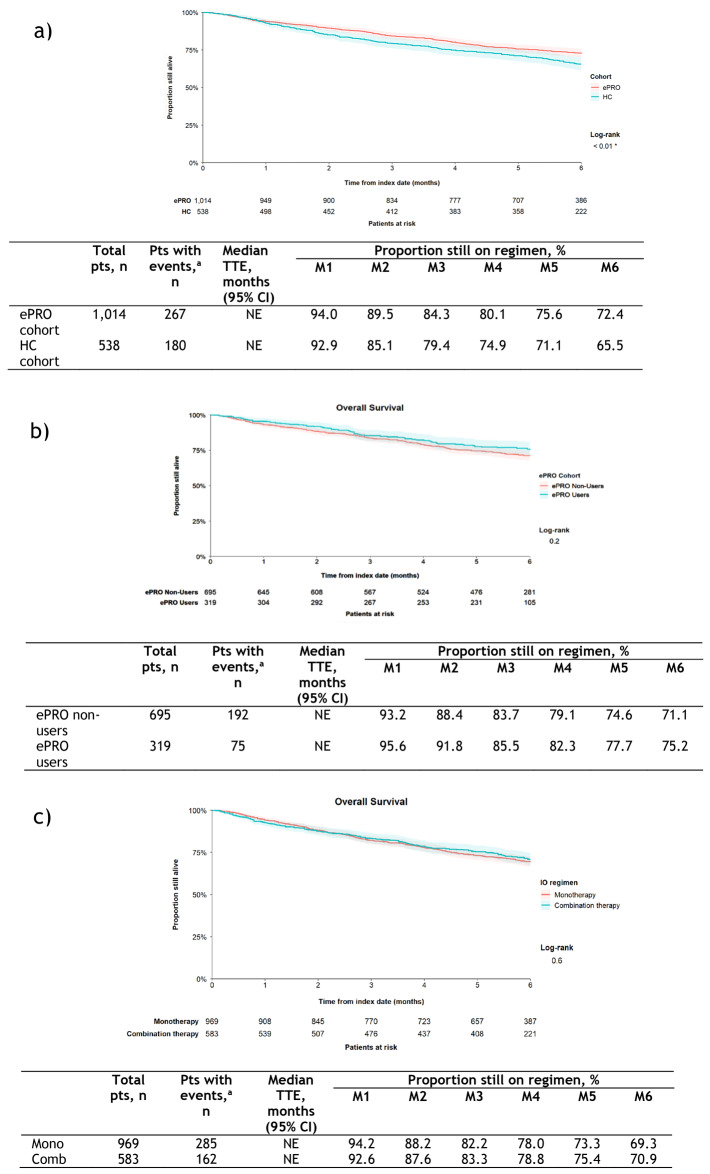
Estimated OS. **a** HC vs ePRO cohort, **b** ePRO cohort users vs non-users, and c) monotherapy vs combination therapy subgroups for first IO regimen. ^a^Patients who did not die or had unknown death date were censored at date of last contact with TO). Kaplan-Meier curves were truncated at 6 months from the index date, the maximum observation period. Shaded areas around the curves represent 95% CIs. *CI* confidence interval; *Comb* combination therapy; *ePRO* electronic patient-reported outcome; *HC* historical control; *IO* immuno-oncology; *M* month; *Mono* monotherapy; *NE* not estimable; *OS* overall survival; *Pts* patients; *TO* Tennessee oncology; *TTE* time to event

The estimated OS rate at 6 months was greater in the ePRO cohort than the HC cohort (72.4 vs 65.5%; *p* < 0 .01); median OS was NE in both cohorts (Fig. 5a). Multivariable regression confirmed longer OS in the ePRO vs HC cohort (Supplementary Table S2), with significant effects also seen for age at index date, gender, index cancer, and index cancer stage at diagnosis.

Within the ePRO cohort, the estimated OS at 6 months was similar for ePRO users and non-users (75.2 vs 71.1%; *p* = 0.2); median OS was NE in both groups (Fig. 5b). Multivariable regression showed no difference in length of OS in ePRO users vs non-users (Supplementary Table S2); significant effects were seen for age at index date and index cancer stage at diagnosis.

In the subgroup analysis of all patients receiving monotherapy vs all those receiving combination therapy for first IO regimen there was no difference in estimated OS rate at 6 months (69.3 vs 70.9%; *p* = 0.6); median OS was NE in both subgroups (Fig. 5c). Median time to end of follow-up was 179.0 days (range 0.0–184.0) in the monotherapy subgroup and 175.0 (range 0.0–184.0) in the combination therapy subgroup. Multivariable regression confirmed there was no difference in survival in the monotherapy vs combination therapy subgroups (Supplementary Table S2); significant effects were seen for age at index date, sex, index cancer, and index cancer stage at diagnosis.

### EPRO platform usage

In the ePRO users group, use of the platform by both providers and patients was durable over the 6-month study period. There was no change in the total number of questionnaires sent to patients by providers from the first time period (Months 1–3) to the second time period (Months 4–6; Table 4). There was a small decrease in the number of questionnaires answered per patient and a small increase in the total number of questionnaires expired per patient.

**Table 4:** Use of ePRO platform in ePRO users subgroup

Parameter	Months 1–3 after index date^a^ (n = 259)	Months 4–6 after index date^a^ (n = 120)
Questionnaires		
Total number of questionnaires sent per patient		
Mean (SD)	6.5 (3.0)	6.5 (3.3)
Median	6.0	6.0
Total number of questionnaires answered per patient		
Mean (SD)	3.7 (2.3)	3.2 (2.1)
Median	4.0	3.0
Answered by the patient		
Mean (SD)	3.1 (2.1)	2.5 (2.1)
Median	3.0	2.0
Answered by the caregiver		
Mean (SD)	0.3 (0.9)	0.2 (1.0)
Median	0.0	0.0
Answered by the clinic		
Mean (SD)	0.3 (0.7)	0.5 (1.0)
Median	0.0	0.0
Total number of questionnaires expired per patient		
Mean (SD)	2.8 (2.4)	3.3 (2.5)
Median	2.0	3.0
Patient/caregiver-reported symptoms		
Number of symptoms per patient		
Mean (SD)	0.7 (1.4)	0.4 (1.1)
Median	0.0	0.0
Number of reported symptoms, n (%)		
0	189 (73.0)	99 (82.5)
1–2	35 (13.5)	12 (10.0)
3–4	30 (11.6)	7 (5.8)
5–6	4 (1.5)	1 (0.8)
7+	1 (0.4)	1 (0.8)
Number of severe^b^ symptoms per patient		
Mean (SD)	0.1 (0.4)	0.0 (0.2)
Median	0.0	0.0
Number of reported severe^b^ symptoms, n (%)		
0	241 (93.1)	116 (96.7)
1–2	16 (6.2)	4 (3.3)
3–4	2 (0.8)	0 (0.0)
5–6	0 (0.0)	0 (0.0)
7+	0 (0.0)	0 (0.0)
Symptom alerts		
Proportion of patients with any alerts, n (%)	29 (11.2)	9 (7.5)
Number of alerts per patient		
Mean (SD)	0.2 (0.6)	0.1 (0.4)
Median	0.0	0.0
Proportion of patients in each outcome category,^c,d^ n (%)		
Provider consulted	6 (20.7)	3 (33.3)
Hospital admission	1 (3.4)	0 (0.0)
Resolved on phone/message	26 (89.7)	5 (55.6)
Scheduled visit	3 (10.3)	1 (11.1)
Follow-up required^e^	4 (13.8)	2 (22.2)
Non-clinical call/message	0 (0.0)	0 (0.0)
No follow-up needed	0 (0.0)	0 (0.0)
Outcome per alert^f^		
Number of alerts	61	37
Proportion of alerts in each outcome category,^c^ n (%)		
Provider consulted	9 (14.8)	3 (8.1)
Hospital admission	1 (1.6)	0 (0.0)
Resolved on phone/message	49 (80.3)	29 (78.4)
Scheduled visit	4 (6.6)	1 (2.7)
Follow-up required^e^	10 (16.4)	3 (8.1)
Non-clinical call/message	1 (1.6)	3 (8.1)
No follow-up needed	0 (0.0)	0 (0.0)

^a^Month 1 was defined as starting on the index date; for the 3-month period analyses, results were reported among ePRO users who were followed for the entirety of each assessment period

^b^Degree of severity was reported by patients or caregivers via the ePRO platform. Of the 14 patient- or caregiver-reported symptoms, 11 could be reported as severe

^c^Each alert may result in more than one outcome. The proportion of patients with any alerts in each outcome category was reported

^d^Denominator is the number of patients with any alert

^e^“Follow-up required” included the case outcome options of “additional follow-up required,” “needs follow-up,” and “left message.”

^f^ePRO users were not required to have complete follow-up in each time period assessed for alert outcomes

*ePRO* electronic patient-reported outcome; *SD* standard deviation

The number of patient/caregiver-reported symptoms decreased slightly in the second time period compared with the first period (mean 0.4 [SD 1.1] vs 0.7 [1.4], respectively; Table 4). However, most patients did not report any symptoms. The number of severe symptoms reported also decreased from the first time period to the second, with the frequency of 1–2 reported severe symptoms dropping from 6.2 to 3.3%, although most patients did not report any severe symptoms. From the first 3-month period to the second 3-month period, there was a reduction in the proportion of patients with any symptom alerts (11.2 vs 7.5%) and in the mean number of alerts (0.2 [SD 0.6] vs 0.1 [0.4]; Table 4). Fewer alerts were issued in the second 3-month period than the first 3-month period. In both time periods, the most frequent alert outcome was “resolved on phone/message”.

## Discussion

This observational, non-interventional study was, to our knowledge, the first to evaluate the use of an ePRO platform and its impact on treatment patterns, particularly DoT, in patients with solid tumors receiving IO therapy in community practice. Treatment patterns for the index IO regimen differed between the HC and ePRO cohorts in that most patients in the HC cohort received monotherapy whereas in the ePRO cohort approximately half received monotherapy and half combination therapy. Consequently, use of chemotherapy and targeted therapy in the index IO regimen was more frequent in the ePRO cohort. These differences likely reflect changes in standard of care for solid tumors over the period of ePRO platform implementation, with the approval of several targeted therapies (including combinations) and increased used of IO-chemotherapy, IO-targeted therapy, and IO-IO combinations, which have shown improved outcomes compared with IO monotherapy, chemotherapy, and targeted monotherapy [28–32].

Index IO regimen DoT was longer in the ePRO vs HC cohort in this study, and multivariable regression analysis confirmed a lower risk of the index IO regimen being discontinued in the ePRO vs HC cohort. OS was also longer in the ePRO cohort than the HC cohort (although median OS was not reached in either cohort), with multivariable analysis confirming longer survival in the ePRO vs HC cohort. Baseline differences between the ePRO and HC cohorts in age and disease stage, which could potentially impact on DoT and OS outcomes, were adjusted for in the multivariable analysis. However, it is unclear whether use of the ePRO platform resulted in the improvements in DoT and OS given the voluntary nature of real-world adoption of the platform, and more importantly, the differences in treatment patterns between the two cohorts, reflecting broader changes in standards of care, which are expected to play a key role in the differing clinical outcomes. In the subgroup analysis comparing ePRO users and ePRO non-users, DoT for the index IO regimen and OS did not differ between groups, supporting the possibility that broader changes in clinical practice occurring after ePRO platform implementation may have been the reason for the improvements in the ePRO cohort, although the smaller sample sizes of ePRO users and ePRO non-users could be a confounding factor. However, the subgroup analysis comparing all patients who received monotherapy vs all those who received combination therapy for the first IO regimen showed no differences in DoT or estimated OS rate over 6 months, suggesting that the differences in mono- vs combination therapy patterns between the ePRO and HC cohorts had minimal impact on these outcomes.

Among patients in the ePRO cohort, uptake of the ePRO platform was 31%. This is comparable with two recent US studies reporting ePRO use in single institutions (initial response rates of 10–20% and 37%) [33, 34]. Lack of computer literacy and online access are likely to be significant factors affecting patient engagement with ePROs in the real world, and lower education level and non-working status are also possible barriers [15]. In our study, patient/caregiver access to suitable electronic devices and frequency of email use were unknown in most cases, but as might be expected, confirmed patient/caregiver access to a suitable device and frequency of patient email use were higher in ePRO users than in non-users. Social determinants also appeared to influence use of the platform, with greater proportions of White patients and patients having a college/higher-level education in the users group vs non-users, although information on education level was unknown in a substantial proportion of patients.

Implementation of the ePRO platform resulted in several learnings, some of which have already been addressed at TO to improve future uptake of the platform. An organizational commitment to understanding and navigating hurdles was required, eg, transitioning from the previous patient portal and telephone triage software to the new integrated platform needed substantial effort from operations, information technology, and clinical informatics teams to minimize disruption to established clinic workflows. Ensuring consistency in TO branding for all communications was also important to assure patients that they were legitimate. Questionnaires were based on the PRO-Common Terminology Criteria for Adverse Events typically used in clinical trials, but over time we realized that a better approach would be to phrase questions in an accessible way so they can be easily answered by the wide range of patients seen in our clinics. Despite increased reliance on technology during the COVID-19 pandemic, a substantial proportion of TO patients cannot/choose not to use an online care coordination platform. Lack of access to adequate internet or technology, lack of digital skills, and wariness around sharing personal information electronically continue to remain challenges for patients and, ultimately, for their care team.

In those patients opting to use the platform (ePRO users group), its use was durable over the 6-month period, with no change in total number of questionnaires sent to patients from the first time period (Months 1–3) to the second period (Months 4–6). A small decrease in the number of questionnaires answered per patient and a small increase in the total number of questionnaires expired per patient was observed, which is in line with findings on longitudinal PRO completion [35]. The platform was successfully employed by ePRO users to report their symptoms, although in the second time period (Months 4–6) there was a slight decrease in the number reporting symptoms and in the number of symptom alerts per patient compared with the first time period; it is not possible to determine the reasons for this, which may include better management of symptoms or a decrease in use of the platform. The majority of symptom alerts had the outcome of “resolved on phone/message.” Previous research has suggested adherence to PRO completion ranges from 50% to over 80%, with longitudinal PROs decreasing over time [36]. Our study had a completion rate of 31%, suggesting a need to improve patient adherence to PRO completion. Influences on factors that improve patient adherence to PRO completion are not clear from currently available evidence, although clinician and administrative engagement in the reporting of PROs has been suggested as a factor to enhance PRO completion [37]. Research has also shown that patients who complete PROs tend to have better functional capacity, meaning missing data may indicate worsening health, among other factors [38].

The strength of the study was the collection of data from the EMR database of one of the largest community-based cancer care practices in the US, which captured detailed information for a large number of patients with a range of solid tumors receiving IO therapies.

The main limitation was that inherent to chart abstraction studies, ie, missing, incomplete, or inaccurate data entries; however, such cases were queried with the treating TO physician where possible. Other limitations include the possibility that the ePRO platform may have been implemented differently for different patients or may have changed over time. Furthermore, it is challenging to separate the impact of ePRO platform implementation from other changes that occurred during the same period both at the individual patient level and the institutional management level, and from broader circumstances that impacted patient care and/or outcomes (eg, changes in standard of care, the COVID-19 pandemic). The time period studied was limited in an attempt to mitigate this. However, further studies are necessary to separate the effects of the ePRO platform from other changes in patient care.

## Conclusion

In summary, improvements in DoT and OS were seen after implementation of the ePRO platform in both ePRO users and non-users. The implementation coincided with changes in the standard of care for solid tumors, which was reflected by the differences in treatment patterns between the HC and ePRO cohorts, although subgroup analysis suggested that differences based on use of monotherapy vs combination therapy had minimal impact on these outcomes. Voluntary patient participation in ePRO use outside the clinic is likely to confound results in the evaluation of real-world outcomes, and further investigation is warranted to determine whether the platform played a role in the improvements observed. The use of ePROs has the potential to facilitate improved care coordination and may enable patients to remain on IO therapy for longer. Our ultimate vision at TO is a comprehensive platform that touches on multiple aspects of clinical care. Careful, continuous evaluation of the implementation process and ongoing monitoring and adjustment of this innovation will be crucial to success.

### Electronic supplementary material

Below is the link to the electronic supplementary material.

Supplementary Material 1

## Data Availability

The datasets generated and analyzed during the current study are not publicly available because they contain information that could compromise the privacy of the research participants.

## Abbreviations

CI: Confidence interval
DoT: Duration of therapy
EMR: Electronic medical record
ePRO: Electronic patient-reported outcome
HC: Historical control
HCRU: Healthcare resource utilization
HR: Hazard ratio
IO: Immuno-oncology
irAEs: Immune-related adverse events
NE: Not estimable
NSCLC: Non-small cell lung cancer
OS: Overall survival
PRO-CTCAE: Patient reported outcome-common terminology criteria for adverse events
SD: Standard deviation
TO: Tennessee Oncology
