# Supplementary material for: Real-world use and clinical impact of an electronic patient-reported outcome tool in patients with solid tumors treated with immuno-oncology therapy
Source: J Patient Rep Outcomes. 2024 Feb 28;8:23. doi: 10.1186/s41687-024-00700-4 (PMC10899997; doi:10.1186/s41687-024-00700-4)
Supplement: Supplementary file 1 — Supplementary Material 1 [file 41687_2024_700_MOESM1_ESM.docx]

**Supplementary information**

**Supplementary Table S1. Demographic, disease, and treatment characteristics in the monotherapy and combination therapy subgroups for first IO regimen**

| **Parameter** | **First IO regimen: monotherapy (n = 969)** | **First IO regimen: combination therapy (n = 583)** | ***P* value^a^** |
| --- | --- | --- | --- |
|  |  |  |  |
| Cohort, n (%)  HC  ePRO | 442 (45.6)  527 (54.4) | 96 (16.5)  487 (83.5) | < 0.001 |
| Age at index date, years, mean (SD) | 70.4 (11.0) | 66.6 (10.1) | < 0.001 |
| Female, n (%) | 367 (37.9) | 243 (41.7) | 0.152 |
| Race, n (%)  White  Black or African American  Asian  American Indian or Alaska Native  Native Hawaiian or Other Pacific Islander  Mixed race  Unknown^b^ | 867 (89.5)  77 (7.9)  4 (0.4)  3 (0.3)  2 (0.2)  1 (0.1)  16 (1.7) | 514 (88.2)  57 (9.8)  2 (0.3)  3 (0.5)  0 (0.0)  0 (0.0)  8 (1.4) | 0.475  0.250  1.000  0.678  0.531  1.000  0.832 |
| Ethnicity, n (%)  Hispanic or Latino  Not Hispanic or Latino  Unknown | 7 (0.7)  877 (90.5)  85 (8.8) | 5 (0.9)  510 (87.5)  68 (11.7) | 0.160 |
| Type of insurance,^c^ n (%)  Non-risk-share contracts  Risk-share contracts^d^ | 567 (58.5)  402 (41.5) | 387 (66.4)  196 (33.6) | < 0.01 |
| Highest level of education,^c^ n (%)  High school or less  College  Graduate degree  Unknown | 156 (16.1)  85 (8.8)  25 (2.6)  703 (72.5) | 165 (28.3)  57 (9.8)  22 (3.8)  339 (58.1) | < 0.001 |
| Marital status,^c^ n (%)  Married  Divorced  Widowed  Single  Separated  Unknown | 557 (57.5)  132 (13.6)  162 (16.7)  110 (11.4)  1 (0.1)  7 (0.7) | 350 (60.0)  68 (11.7)  68 (11.7)  84 (14.4)  7 (1.2)  6 (1.0) | < 0.001 |
| Living arrangements,^c^ n (%)  With spouse  Alone  With child  With relatives  Care facility  Other  Unknown | 539 (55.6)  212 (21.9)  85 (8.8)  58 (6.0)  13 (1.3)  38 (3.9)  24 (2.5) | 347 (59.5)  106 (18.2)  36 (6.2)  44 (7.5)  3 (0.5)  36 (6.2)  11 (1.9) | < 0.05 |
| Index cancer,^e^ n (%)  NSCLC  Melanoma  Renal cell carcinoma  Head and neck cancer  Bladder cancer | 603 (62.2)  169 (17.4)  47 (4.9)  85 (8.8)  65 (6.7) | 445 (76.3)  56 (9.6)  61 (10.5)  11 (1.9)  10 (1.7) | < 0.001 |
| Stage of index cancer at diagnosis, n (%)  Stage I  Stage II  Stage III  Stage IV  Unknown | 67 (6.9)  102 (10.5)  330 (34.1)  409 (42.2)  61 (6.3) | 35 (6.0)  37 (6.3)  119 (20.4)  360 (61.7)  32 (5.5) | < 0.001 |
| Progression since diagnosis,^f^ among patients diagnosed with stage I to III, n (%)  Yes, metastatic recurrence  Yes, local/regional recurrence  No  Unknown | 221 (44.3)  75 (15.0)  201 (40.3)  2 (0.4) | 97 (50.8)  19 (9.9)  73 (38.2)  2 (1.0) | 0.135 |
| Number of lines of prior therapy for index cancer,^g,h^ mean (SD) | 0.5 (0.6) | 0.1 (0.4) | < 0.001 |
| Index IO therapy,^e^ n (%)  Atezolizumab  Avelumab  Durvalumab  Ipilimumab  Nivolumab  Nivolumab + ipilimumab  Pembrolizumab | 58 (6.0)  1 (0.1)  155 (16.0)  19 (2.0)  298 (30.8)  0 (0.0)  438 (45.2) | 11 (1.9)  1 (0.2)  67 (11.5)  0 (0.0)  9 (1.5)  106 (18.2)  389 (66.7) | < 0.001 |
| Time to the end of follow-up (days), median (range) | 179.0  (0–184.0) | 175.0  (0–184.0) | 0.936 |
| Mortality during the study follow-up, n (%)  Alive  Deceased  Unknown | 663 (68.4)  289 (29.8)  17 (1.8) | 411 (70.5)  169 (29.0)  3 (0.5) | 0.090 |
| Reason for the end of follow-up (earliest event),^i^ n (%)  Last contact with TO  6-month follow-up  Clinical trial enrollment  Death | 496 (51.2)  437 (45.1)  25 (2.6)  11 (1.1) | 339 (58.1)  219 (37.6)  18 (3.1)  7 (1.2) | < 0.05 |

^a^Statistical comparison performed for continuous variables using Wilcoxon rank-sum test and for categorical variables using chi-squared test or Fisher’s exact tests if expected counts < 10.

^b^Patient declined, or otherwise not documented/unknown.

^c^Collected from the EMR on the date of abstraction and may not reflect the status at the index date.

^d^Risk-share contracts include Medicare, Aetna, and Cigna (for patients who initiated IO therapy after Apr 01, 2020).

^e^The index cancer was defined as the cancer associated with the index IO therapy and the index IO therapy was defined as the IO therapy/therapies initiated on the index date.

^f^Progression reported from diagnosis until end of follow-up, as documented by the treating physician was assessed among the 690 patients diagnosed with stage I, II, or III for their index cancer.

^g^Summary statistics for the number of lines of prior therapy were assessed among patients with known information.

^h^Prior lines of therapy before IO initiation may have occurred at TO or another facility and patients may have had more than one type of therapy.

^i^Reason for end of follow-up was defined as the earliest of the following events, if applicable: 6 months after IO initiation, death, pregnancy, clinical trial enrollment, or last contact with TO.

EMR, electronic medical record; ePRO, electronic patient-reported outcome; HC, historical control; IO, immuno-oncology; NSCLC, non-small cell lung cancer; SD, standard deviation; TO, Tennessee Oncology.

**Supplementary Table S2. Multivariable fitted Cox proportional hazards model for OS**

| **Cohort comparison** |  | **Variate** | **HR (95% CI)** | ***P* value** |
| --- | --- | --- | --- | --- |
| ePRO vs HC |  | Cohort (ePRO vs HC)  Age at index date (years)  Male vs female  White vs non-White  Index cancer vs melanoma^a^  NSCLC  Other  Stage of index cancer at diagnosis vs stage I  Stage II  Stage III  Stage IV  Unknown | 0.82 (0.68–1.00)  1.02 (1.01–1.03)  1.27 (1.04–1.54)  1.09 (0.80–1.47)  1.50 (1.07–2.10)  1.51 (1.04–2.19)  1.04 (0.62–1.73)  0.76 (0.49–1.18)  1.61 (1.08–2.42)  1.00 (0.56–1.78) | < 0.05  < 0.001  < 0.05  0.586  < 0.05  < 0.05  0.882  0.215  < 0.05  0.996 |
| ePRO users vs ePRO non-users |  | Cohort (ePRO users vs ePRO non-users)  Age at index date (years)  Male vs female  White vs non-White  Insurance type (RSC vs non-RSC)  Index cancer vs melanoma^a^  NSCLC  Other  Stage of index cancer at diagnosis vs stage I  Stage II  Stage III  Stage IV  Unknown | 0.82 (0.62–1.08)  1.02 (1.01–1.03)  1.17 (0.90–1.50)  1.06 (0.72–1.55)  1.04 (0.80–1.35)  1.22 (0.78–1.92)  1.41 (0.87–2.28)  1.01 (0.53–1.91)  0.55 (0.32–0.95)  1.57 (0.97–2.56)  0.94 (0.46–1.90) | 1.52  < 0.01  0.235  0.786  0.765  0.377  0.168  0.986  < 0.05  0.069  0.864 |
| Monotherapy vs combination therapy |  | Regimen (monotherapy vs combination therapy)  Age at index date (years)  Male vs female  White vs non-White  Insurance type (RSC vs non-RSC)  Index cancer vs melanoma^a^  NSCLC  Other  Index year (n [%]) vs 2017  2018  2019  2020  Stage of index cancer at diagnosis vs stage I  Stage II  Stage III  Stage IV  Unknown | 1.09 (0.88–1.35)  1.02 (1.01–1.03)  1.26 (1.04–1.54)  1.10 (0.81–1.49)  0.96 (0.79–1.17)  1.52 (1.09–2.14)  1.50 (1.03–2.18)  0.99 (0.67–1.47)  0.79 (0.58–1.07)  0.86 (0.69–1.07)  1.04 (0.62–1.72)  0.75 (0.48–1.16)  1.62 (1.08–2.43)  1.00 (0.56–1.79) | 0.413  < 0.001  < 0.05  0.546  0.676  < 0.05  < 0.05  0.965  0.121  0.185  0.895  0.194  < 0.05  0.996 |

^a^Melanoma was selected as the reference by the analysis software (first tumor when listed alphabetically).

CI, confidence interval; ePRO electronic patient-reported outcome; HC, historical control; HR, hazard ratio; NSCLC, non-small cell lung cancer; OS, overall survival; RSC, risk-share contract.
